# An Amino Acid Signature Associated with Obesity Predicts 2-Year Risk of Hypertriglyceridemia in School-Age Children

**DOI:** 10.1038/s41598-017-05765-4

**Published:** 2017-07-17

**Authors:** Sofia Moran-Ramos, Elvira Ocampo-Medina, Ruth Gutierrez-Aguilar, Luis Macías-Kauffer, Hugo Villamil-Ramírez, Blanca E. López-Contreras, Paola León-Mimila, Joel Vega-Badillo, Roxana Gutierrez-Vidal, Ricardo Villarruel-Vazquez, Erandi Serrano-Carbajal, Blanca E Del-Río-Navarro, Adriana Huertas-Vázquez, Teresa Villarreal-Molina, Isabel Ibarra-Gonzalez, Marcela Vela-Amieva, Carlos A. Aguilar-Salinas, Samuel Canizales-Quinteros

**Affiliations:** 1 Consejo Nacional de Ciencia y Tecnología (CONACYT), Mexico City, Mexico; 2 Unidad de Genómica de Poblaciones Aplicada a la Salud, Facultad de Química, UNAM/Instituto Nacional de Medicina Genómica (INMEGEN), Mexico City, Mexico; 3 Hospital Infantil México Federico Gómez, Mexico City, Mexico; 4 Facultad de Medicina, UNAM, Mexico City, Mexico; 5 Department of Medicine, David Geffen School of Medicine, UCLA, Los Angeles, USA; 6 Laboratorio de Enfermedades Cardiovasculares, INMEGEN, Mexico City, Mexico; 7 Instituto de Investigaciones Biomédicas, UNAM - Instituto Nacional de Pediatría, Mexico City, Mexico; 8 Laboratorio de Errores Innatos del Metabolismo y Tamiz, Instituto Nacional de Pediatría, Mexico City, Mexico; 9 Departamento de Endocrinología y Metabolismo, Instituto Nacional de Ciencias Médicas y Nutrición Salvador Zubirán, Mexico City, Mexico

## Abstract

Childhood obesity is associated with a number of metabolic abnormalities leading to increased cardiovascular risk. Metabolites can be useful as early biomarkers and new targets to promote early intervention beginning in school age. Thus, we aimed to identify metabolomic profiles associated with obesity and obesity-related metabolic traits. We used data from the Obesity Research Study for Mexican children (ORSMEC) in Mexico City and included a case control (n = 1120), cross-sectional (n = 554) and a longitudinal study (n = 301) of 6–12-year-old children. Forty-two metabolites were measured using electrospray MS/MS and multivariate regression models were used to test associations of metabolomic profiles with anthropometric, clinical and biochemical parameters. Principal component analysis showed a serum amino acid signature composed of arginine, leucine/isoleucine, phenylalanine, tyrosine, valine and proline significantly associated with obesity (OR = 1.57; 95%CI 1.45–1.69, *P* = 3.84 × 10^−31^) and serum triglycerides (TG) (β = 0.067, *P* = 4.5 × 10^−21^). These associations were validated in the cross-sectional study (*P* < 0.0001). In the longitudinal cohort, the amino acid signature was associated with serum TG and with the risk of hypertriglyceridemia after 2 years (OR = 1.19; 95%CI 1.03-1.39, *P* = 0.016). This study shows that an amino acid signature significantly associated with childhood obesity, is an independent risk factor of future hypertriglyceridemia in children.

## Introduction

Obesity represents a major global health problem in adults and children. In Mexico, according to the National Health and Nutrition survey 2012, the prevalence of overweight and obesity is 71.2% in adults and 34.3% in children^1^. Pediatric obesity is strongly associated with a number of metabolic abnormalities such as dyslipidemia, insulin resistance and hypertension. All the latter are well known risk factors for cardiovascular disease^2^, the leading worldwide cause of mortality^3^. Low serum high-density lipoprotein cholesterol (HDL-C) levels, elevated low-density lipoprotein cholesterol (LDL-C), high triglycerides (TG) levels, or a combination of the latter are frequent metabolic alterations in childhood obesity^4^. Indeed, it was recently reported that in Mexico almost 70% of obese and 50% of non-obese children have dyslipidemia, where hypertriglyceridemia is the most frequent^5^. Furthermore, lipid levels during childhood are predictive of dyslipidemia and subclinical atherosclerosis in adulthood^6, 7^.

Therefore there is an urgent need to identify risk factors during childhood to prevent future dyslipidemia and ultimately cardiovascular disease in adults^8^. Metabolomics has emerged as a powerful tool to identify novel risk factors for early detection of various health-related traits. Branched-chain (BCAA) and aromatic amino acid (AA) serum levels are increased in obesity in humans and animal models^9, 10^, and have been associated with current and future development of insulin resistance in adults and children^11–13^. Furthermore, a recent meta-analysis confirmed that higher levels of BCAA and AA increase the risk of type 2 diabetes^14^. In addition amino acid profiles have been associated with dyslipidemia in adults^15, 16^. Therefore, the objective of the present study was to identify metabolomic profiles associated with obesity and metabolic traits in Mexican children using both a case-control study and a longitudinal cohort-based approach.

## Methodology

### Study participants

This study was embedded in the “Obesity research study for Mexican children” (ORSMEC), which is a population based study, with the overall aim of identifying risk factors for obesity and metabolic abnormalities in school age children. ORSMEC includes 6–12-year-old children recruited from a summer camp of children of employees of the Mexican Health Ministry (*Convivencia Infantil, Sindicato de la Secretaria de Salud*) and *Hospital Infantil de Mexico* from 2008–2015. All children included were free of chronic medical illness. Parents or guardians of each child signed the informed consent form and children assented to participate. The study was approved by the Ethics Committee of participant institutions; *Instituto Nacional de Medicina Genómica*, *Instituto Nacional de Ciencias Medicas y Nutrición* and *Hospital Infantil de Mexico* and was in accordance with the Helsinki declaration II.

Three different study designs were tested. In the discovery phase, a case-control study for obesity was carried out to seek associations of metabolomic profiles with obesity. This study included 1120 healthy unrelated children [606 normal-weight and 514 obese, classified according to the Center for Disease Control and Prevention (CDC) criteria^17^]. Obesity status was defined as body mass index (BMI) percentile ≥95^th^ for age and gender, while for the normal-weight group children with BMI between the 15^th^ and 75^th^ percentile for age and gender were selected. An independent cross-sectional study was performed including 554 children from ORSMEC recruited between 2013 and 2014, excluding those who were part of the initial case-control study. Finally, a longitudinal cohort was designed to test for metabolomic biomarkers predicting obesity and related metabolic traits. This included children, that attended two times to the summer camp and whose clinical data and biological samples, with a 2-year separation, were available (n = 301).

### Anthropometric and clinical parameters

Anthropometric measurements were determined following the procedures recommended by Lohman *et al*.^18^ and included weight, height, waist and hip circumferences. All instruments were calibrated following the standard methods of the manufacturers. Blood pressure (BP; mmHg) was measured twice at sitting position using an automatic manometer (Microlife), and the average of both measurements was used for the analyses. Body fat mass percentage was obtained by bioelectrical impedance analysis (Quantum X Body Composition Analyzer, RJL Systems). Center for Disease Control 2000 growth charts were used as reference to determine BMI percentiles^17^. As previously mentioned, obesity status was defined as body mass index (BMI) percentile ≥95^th^, whereas overweight was defined between 85^th^ and 95^th^ percentile, normal-weight between the 5^th^ and 85^th^ percentile and underweight < 5^th^ BMI percentile for age and gender. National Heart, Lung, and Blood Institute reference data were used to determine BP percentiles based on height, age and gender^19^. Hypertriglyceridemia was defined as serum triglyceride levels ≥130 mg/dL as suggested by the Expert Panel on Integrated Guidelines for Cardiovascular Health Risk Reduction in Children and Adolescents^20^.

### Blood sampling and biochemical analyses

Five mL blood samples were drawn after 8–12 hours of fasting. For serum samples a 30-min period was allowed for clotting before serum separation and then stored at −80 °C until further analysis. Serum levels of glucose, creatinine, uric acid, total cholesterol, HDL cholesterol, LDL cholesterol, TG, aspartate aminotransferase (AST), alanine aminotransferase (ALT), gamma glutamyl transpeptidase (GGT), Apo B and C-reactive protein (CRP) were measured with commercially available standardized methods (UNICEL DxC600, Beckman coulter). Insulin was determined using an Access 2 Immunoassay System (Beckman coulter). Insulin sensitivity was indirectly estimated with the homeostasis model assessment for insulin resistance (HOMA-IR).

### Metabolomics analysis

We used a targeted metabolomics approach by electrospray tandem mass spectrometry (Quattro Micro API tandem MS) that includes metabolites previously associated with obesity and obesity-related traits and that is also useful to assess amino acid and fatty acid metabolism in a high number of samples^9^. Serum concentrations of 11 amino acids, free carnitine and 30 acylcarnitines were measured with a commercial kit (NeoBase Non-derivatized MS/MS kit; PerkinElmer Waltham Massachusetts) using multiple reaction monitoring (MRM) mode. The assay was performed according to the manufacturer’s instructions. Briefly, fasting serums samples (previously stored at −80 °C) were applied to filter paper cards (Whatman 903^TM^, GE Healthcare Bio-Sciences Corp. Westborough, MA) and dried for 3 hours. For every sample a 3mm-diameter disk was punched with an automatic device into a 96-well sample plate where 190 µL of extraction solution containing a mixture of 22 stable isotope-labeled internal standards were added (Supplemental Table 1). The plate was covered with aluminum foil, incubated with agitation (30 °C at 650 × g for 30 min) and placed in a 2777 C Waters auto-sampler (Waters Corp., Milford, MA). An HPLC pump (Waters 1525μ) was used for flow injection analysis (FIA). Thirty μL of sample extracts were directly injected at a flow rate of 1.5 mL/min and in an analysis time of 1.5 min. A blank sample (extraction solution with internal standards) was included in each plate. Low and high analytical controls as well as a quality control sample (pool of all serum samples) were included in each plate in triplicate. Metabolites were quantified by reference to appropriate internal standards. Intra- and inter-plate variation coefficients were calculated based on repetitive measurements of the quality control sample. Leucine and isoleucine are reported as a single analyte because they are not resolved by the MS/MS method. Inter and intra-assay variation coefficients for arginine, leucine/isoleucine, valine, phenylalanine, tyrosine and proline ranged from 5 to 9%.

### Statistical Analysis

In the case-control study, differences between groups were assessed using Mann-Whitney-Wilcoxon Test. In the discovery phase, a principal component analysis (PCA) was performed on the 42 metabolites using Facto-MineR in R package. This package performs unit variance scaling of metabolites as data pre-treatment prior to PCA^21^. Components with eigenvalues >1.0 were retained and metabolites with component loading values higher than >0.5 were retained for a given component. Logistic regression models were used to test the effect of the principal components (PCs) on the risk for obesity, while linear regression was used to test the association with clinical and biochemical variables. The degree of multicollinearity among metabolites was estimated with the Variance Inflation Factor (VIF).

To test the findings in the cross-sectional and longitudinal studies a combined index of the amino acids represented in principal component 2 (PC2) was generated. Cohort Z-score values were calculated for each normalized metabolite in all subjects. The sum of amino acid Z-scores represented in PC2 (PC2-Z) was tested for associations with metabolic traits by linear regression models. In the longitudinal cohort, paired sample Wilcoxon signed-rank test was used to compare clinical and biochemical variables between baseline (T = 0) and 2 year follow up measurements (T = 1). The association of the PC2-Z with hypertriglyceridemia after 2 years was assessed with multivariate logistic regression analysis, using stepwise modeling to determine the effect of other risk factors or potential confounders (gender and baseline age, BMI percentile, TG and HOMA-IR). C statistics was used to test the ability of several models to predict hypertriglyceridemia, by calculating the area under the receiver operating characteristic curve (AUC). Furthermore, the contribution of the amino acid component was assessed by the integrated discrimination index (IDI) and the net reclassification improvement (NRI) method, which evaluates the proportion of subjects moving accurately or inaccurately from one risk category to another, after adding the predictor of interest into the model^22^. For NRI calculation, participants were assigned to one of four categories reflecting their risk of 2-year hypertriglyceridemia based on each model (<5, 5 to <10, 10–20, and >20%). Model calibration was tested by the Hosmer-Lemeshow χ^2^ test. All non-normally distributed clinical variables were transformed using the Box-Cox method before testing associations. This algorithm transforms data into a normal shape by using the parameter λ. Logistic and linear regression models were adjusted for age, gender and sampling calendar year, while associations with biochemical variables were also adjusted for BMI percentile. The resulting *P*-value was corrected for multiple comparisons with false discovery rate (FDR) method^23^. Statistical significance was set at *P* < 0.05.

## Results

### Case control study

Anthropometric and biochemical characteristics in normal weight and obese children are described in Supplemental Table 2. Body fat percentage and all clinical parameters excluding lean mass percentage and HDL-C levels were significantly higher in obese than in normal weight children. To analyze the metabolomics profile, we used PCA that cluster metabolites into highly correlated species. Seven components including 37 metabolites showed eigenvalues >1, which explained 69% of the variance in metabolites. Component 7 was excluded from the analysis since no metabolites had loading values greater that 0.5 (results not shown). Six components explaining the 66.6% of variance in metabolites were retained for further analysis. The constituents of each component are shown in Table 1.

**Table 1:** Principal component analysis of 42 metabolites and associations of clusters with obesity in a case-control cohort of Mexican school-age children.

	Description	Component metabolites	Eigenvalue	Percentage of variance	Cumulative percentage of variance	Obesity		Percentage of variance explaining obesity
						OR	*P* value	
PC1	Miscellaneus	Citrulline, Glycine, Alanine, Leucine + Isoleucine, Methionine, Phenylalanine, Valine, Succinylacetone, Propionylcarnitine C3, Butyryl + isobutyrylcarnitine C4, Tiglylcarnitine C5:1, Hexanoylcarnitine C6, Octanoylcarnitine C8, Octenoylcarnitine C8:1, Hexadecenoylcarnitine C16:1, Hydroxyhexadecenoylcarnitine C16:1-OH, Hydroxypalmitoylcarnitine C16-OH, Decanoylcarnitine C10, Decenoylcarnitine C10:1, Decadienoylcarnitine C10:2, Dodecanoylcarnitine C12, Dodecenoylcarnitine C12:1, Tetradecanoylcarnitine C14, Tetradecenoylcarnitine C14:1, Tetradecadienoylcarnitine C14:2, 3-hydroxytetradecenoylcarnitine C14:OH, Stearylcarnitine C18, Linoleylcarnitine C18:2, Hydroxybutyrylcarnitine C4OH + Malonylcarnitine C3DC, Hydroxyisovalerylcarnitine C5OH + Succinyl + methylmalonylcarnitine C4DC	14.39	33.46	33.46	1.04 (1.00–1.07)	**0.03**	1.03
PC2	Amino acids	Arginine, Leucine + Isoleucine, Phenylalanine, Tyrosine, Valine, Proline	5.20	12.09	45.55	1.57 (1.45–1.69)	**3.84 × 10** ^**−31**^	15.28
PC3	Acycarnitines	Acetylcarnitine C2, Palmitoylcarnitine C16, Stearylcarnitine C18	3.23	7.51	53.06	1.21 (1.11–1.33)	**4.46 × 10** ^**−5**^	0.88
PC4	Shortchain acylcarnitines	Acetylcarnitine C2, Propionylcarnitine C3	2.49	5.78	58.84	0.97 (0.89–1.04)	0.409	0.03
PC5	Medium chain acylcarnitines	Methylglutarylcarnitine C6DC, Dodecanoylacylcarnitine C12	2.13	4.95	63.79	0.53 (0.47–0.60)	**7.63 × 10** ^**−25**^	6.52
PC6	Miscellaneus	Ornithine, Octenoylcarnitine C8:1	1.37	3.18	66.98	0.93 (0.84–1.03)	0.19	0.30

The logistic regression model was adjusted for gender and age.Significant P-values are shown in bold.

Using logistic regression models, we found three components significantly associated with a higher risk of obesity [PC1 (OR = 1.04; 95%CI 1.0–1.07, P = 0.037), PC2 (OR = 1.57; 95%CI 1.46–1.70, *P* = 3.8 × 10^−31^) and PC3 (OR = 1.21; 95%CI 1.11–1.33, *P* = 4.46 × 10^−5^)], while PC5 (OR = 0.53; 95%CI 0.47–0.60, *P* = 7.63 × 10^−25^) was associated with a lower obesity risk. PC2, represented by high serum levels of arginine, leucine/isoleucine, phenylalanine, tyrosine, valine and proline showed the strongest association with obesity and explained 15.3% of the variance (Table 1). Multicollinearity among the amino acids present in PC2 was low to modest (VIF 1.5–6.7), and arginine and proline showed the lowest VIF values (1.5 and 1.9 respectively).

**Figure 1:**
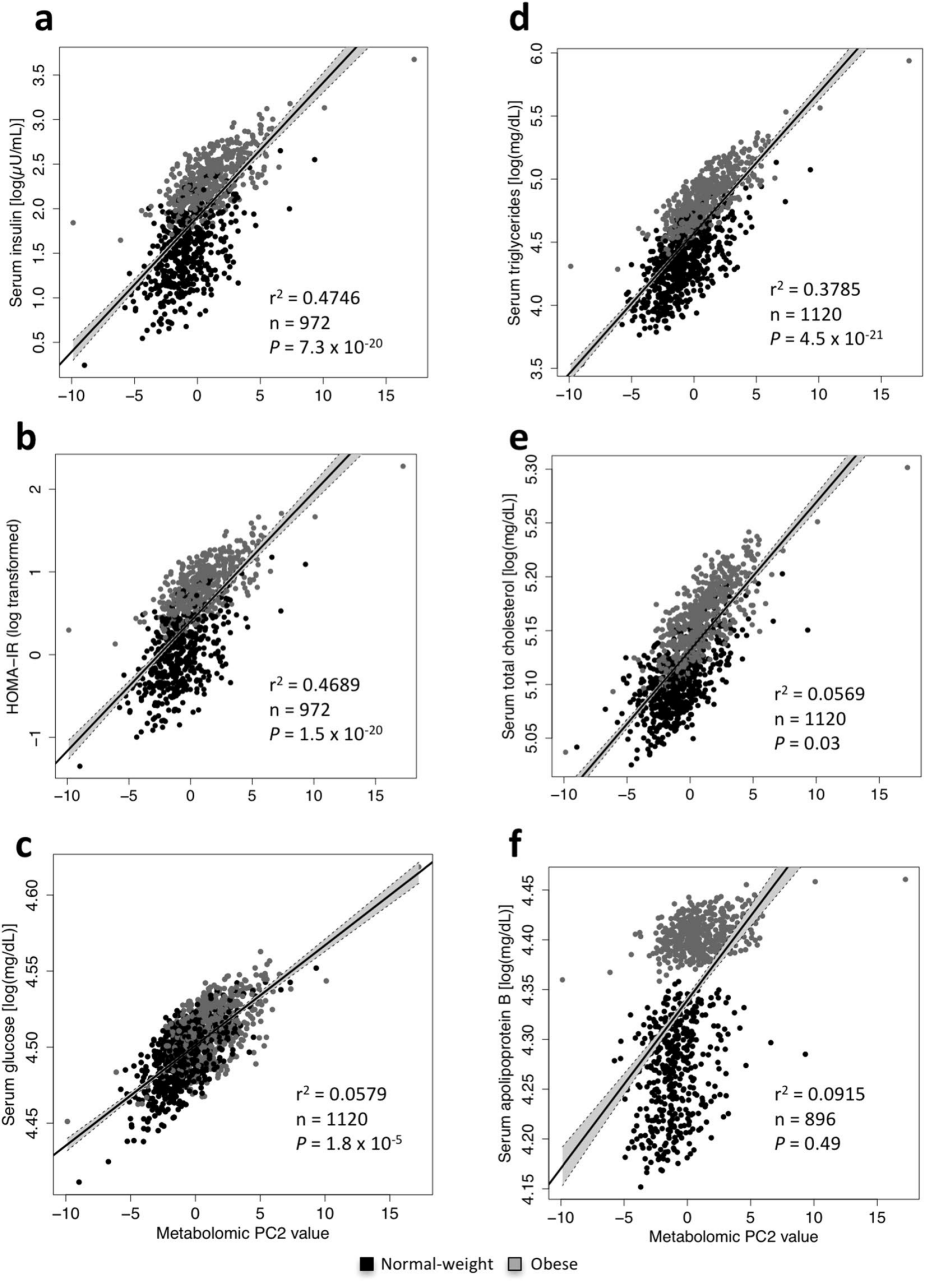
Case-control study. Correlation between PC2 amino acids (arginine, leucine/isoleucine, phenylalanine, tyrosine, valine and proline) and serum insulin (**a**), HOMA-IR (**b**), serum glucose (**c**), serum triglycerides (**d**), serum total cholesterol levels (**e**) and serum ApoB (**f**). The linear regression model was adjusted by age, gender, BMI percentile and sampling calendar year. *P*-values were corrected by FDR.

We then tested whether these PCs were associated with other clinical variables (Supplemental Table 3). The amino acid component (PC2) showed the strongest associations with HOMA-IR, serum insulin, glucose, TG and total cholesterol levels, but not with ApoB (Fig. 1). PC2 was also significantly associated with higher systolic BP percentile. Moreover, PC3, represented by C2 and C16 carnitines, showed positive associations with systolic BP percentile, creatinine, uric acid, AST and GGT levels. Component PC5, negatively associated with obesity, showed no significant associations with biochemical variables (Supplemental Table 3). Because the amino acid component (PC2) explained the highest percentage in obesity variance and was also associated with biochemical variables, we focused on this component to test the findings in a cross-sectional and a longitudinal study

### Cross-sectional study

The cross-sectional study included 554 children with available fasting metabolomics data, and with the following weight status distribution: 7.4% underweight, 51.4% normal weight, 31.9% overweight and 9.2% obese. Their clinical characteristics are summarized in Supplemental Table 4. In consistency with the observations in the case-control study, in this study, PC2-Z was associated with obesity (OR = 1.18; 95%CI 1.05–1.34, *P* = 9.3 × 10^−3^), BMI percentile, fat mass percentage, insulin, and TG and levels (all *P* < 0.05; Supplemental Table 5). PC2-Z was also associated with HDL cholesterol and GGT levels (all *P* < 0.05; Supplemental Table 5). On stratifying by weight status, the association of PC2-Z with serum TG and GGT was nominally significant, and remained close to significance after FDR correction in all groups (*P* < 0.17), except for underweight children (Supplemental Table 6). The associations of PC2-Z with serum insulin and HDL-cholesterol levels did not reach significance in any weight status group after FDR correction (*P* > 0.16; Supplemental Table 6).

### Longitudinal cohort

**Figure 2:**
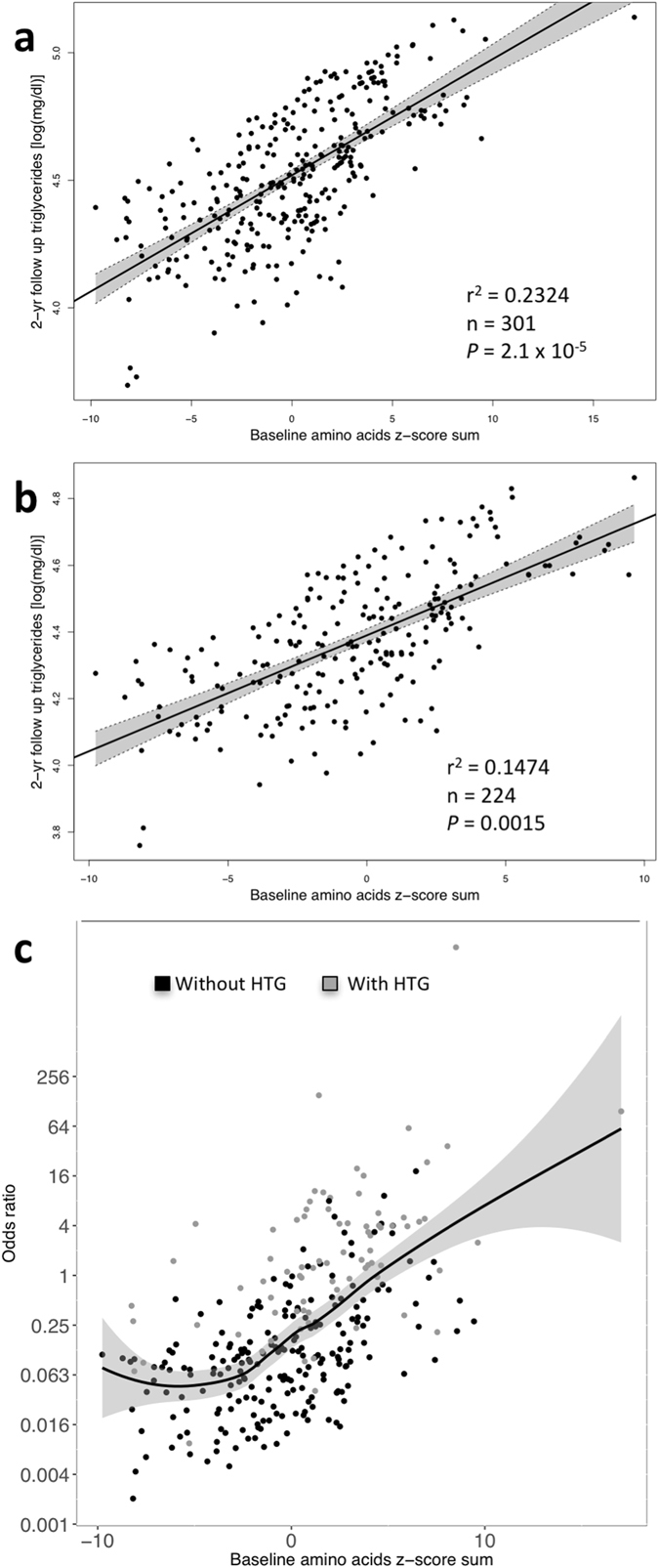
Longitudinal study. Association between z-score sum of baseline amino acids from PC2-Z (arginine, leucine/isoleucine, phenylalanine, tyrosine, valine and proline) and triglycerides after 2 years. (**a**) All individuals. (**b**) Individuals without hypertriglyceridemia (HTG) at baseline. (**c**) Logistic regression model reflecting risk of HTG (with 95% CI) according to PC2-Z and excluding children with HTG at baseline (n = 224). The multivariate model was adjusted by gender, and sampling calendar year as well as baseline age, BMI percentile and serum triglycerides.

The longitudinal cohort included children followed for 2 years and with the following weight status distribution at baseline: 4.9% underweight, 53.8% normal weight, 16.6% overweight and 24.6% obese. Clinical characteristics measured at baseline and after 2 years are described in Supplemental Table 7. Height, body weight, BMI, fat mass percentage and several biochemical variables (insulin, HOMA-IR, uric acid, ALT and GGT) increased significantly over time. In contrast, systolic BP percentile, glucose, creatinine, total cholesterol and LDL cholesterol, showed a slight but significant decrease after the 2-year follow up (Supplemental Table 7). We used linear regression models to assess the associations of baseline amino acids (represented in PC2) with future metabolic traits. Consistently with the case-control and cross-sectional studies, PC2-Z was significantly associated with 2-year BMI percentile (*P* = 1 × 10^−3^), body fat percentage (*P* = 6 × 10^−4^; Supplemental Table 8) and after further adjustment for BMI percentile, with serum TG levels (Fig. 2a). The association with TG remained significant even after excluding children with hypertriglyceridemia at baseline (≥130 mg/dL) (*P* = 1.5 × 10^−3^; Fig. 2b). PC2-Z also showed a significant positive association with serum insulin levels, although lost significance after correcting for multiple tests (Supplemental Table 8).

Because PC2-Z was associated with TG levels in the three studies, we examined whether this component was associated with future development of hypertriglyceridemia. Seventy-seven children (25%) with hypertriglyceridemia at baseline were excluded from the analysis. After 2 years, 32 children developed hypertriglyceridemia. Figure 2c shows that increased levels of the amino acid signature (as represented by the amino acid Z-score sum) showed association with higher odds of hypertriglyceridemia after 2 years. This association remained significant after adjusting for gender and baseline age, as well as potential confounders such as baseline BMI percentile, HOMA-IR and TG (OR = 1.19; 95%CI 1.03–1.39, *P* = 0.016; Table 2). This suggests that PC2-Z is an independent risk factor for hypertriglyceridemia. The area under the receiver operating characteristic (ROC) curve (AUC) for PC2-Z was 0.7067 (95%CI 0.6046–0.8088, *P* < 0.0001), stronger than fat mass percentage, BMI percentile and HOMA-IR (data not shown). Using multivariate logistic regression models, we studied the utility of three different models in the prediction of the development of hypertriglyceridemia over a 2-year period which included: 1) clinical variables (gender, baseline age and BMIp) 2) clinical and biochemical variables (baseline HOMA-IR and TG) 3) clinical and biochemical variables plus the metabolomic signature (Table 3). The three models showed a good fit as evaluated by the Hosmer-Lemeshow test (All *P* > 0.5). The model including clinical and biochemical variables as well as the metabolomic signature showed the highest prediction value (AUC = 0.811 95%CI 0.726–0.897; Table 3). To evaluate the improvement in the prediction risk by adding the metabolomic signature, we calculated the integrated discrimination index (IDI) and the net reclassification improvement (NRI). The addition of the metabolomic signature to the model showed a 7% net reclassification improvement as well as significant IDI (*P* < 0.01: Table 3).

**Table 2:** Associations between baseline amino acid signature and odds of hypertriglyceridemia, adjusting for gender and baseline age, BMI percentile, HOMA and serum TG.

Model	Adjustments	OR	95% Confidence interval		
			Lower CI	Upper CI	*P* value
1	Age and gender	1.25	1.09	1.44	**0.0019**
2	Model 1 + BMI percentile	1.23	1.07	1.42	**0.0040**
3	Model 2 + Baseline HOMA	1.22	1.07	1.42	**0.0040**
4	Model 3 + Baseline serum TG	1.19	1.03	1.39	**0.0160**

All models were adjusted by sampling calendar year. Significant P-values are shown in bold.

**Table 3:** Comparison of models for prediction of development of hypertrigliceridemia over 2 years in children with normal TG levels at baseline.

	AUC (95% CI)	*P value*	NRI	*P value*	IDI	*P value*	H-L	*P value*
Model 1: Gender, age and BMI percentile	0.757 (0.669–0.845)	**1.7 × 10** ^**−6**^					11.49	0.175
Model 2: Model 1 + Baseline HOMA-IR and serum TG	0.796 (0.712–0.880)	**4.7 × 10** ^**−8**^	20%	**0.00045**	6.0%	**0.0058**	10.35	0.242
Model 3: Model 2 + Amino acids Z-score sum	0.811 (0.726–0.897)	**9.4 × 10** ^**−9**^	7%	**0.00356**	4.5%	**0.0047**	6.62	0.578

H-L: Hosmer-Lemeshow χ^2^ statistics, NRI: net reclassification improvement, IDI: integrated discrimination index.

When analyzing amino acids composing PC2-Z individually, phenylalanine showed the strongest association with hypertriglyceridemia risk after adjusting for potential confounders (OR = 2.12; 95%CI 1.24–3.81, *P* = 0.0094; Supplemental Table 9). Moreover, the AUC for phenylalanine was similar to that of the entire amino acid signature (AUC = 0.6901; 95%CI 0.5806–0.7960, *P* < 0.0001).

## Discussion

In the last decades, the prevalence of obesity has increased significantly in children. This epidemic has resulted in a large population of children and adolescents with obesity-associated metabolic complications such as dyslipidemia^4^, an independent risk factor for atherosclerosis. Early identification of children with a high risk of developing dyslipidemia is crucial to establish preventive measures to decrease cardiovascular risk in adulthood. We used a targeted metabolomics approach to identify metabolomic profiles associated with obesity, metabolic complications and increased cardiovascular risk. In the case-control study, the major metabolomic amino acid signature (PC2: arginine, leucine/isoleucine, phenylalanine, tyrosine, valine and proline) was associated with obesity, insulin resistance and lipid levels. This is consistent with previous reports where BCAA (leucine, isoleucine and valine) and AA (phenylalanine, tyrosine, tryptophan) were associated with obesity and insulin resistance in both adults and children^12, 15, 24, 25^. Although less consistently, high serum levels of proline and arginine have been observed in obese and hyperlipidemic adults^11, 26^. In our study, because VIF values for the latter amino acids were low, collinearity is unlikely to explain their association with obesity risk. However, further studies are needed to confirm whether proline and arginine are in fact associated with a higher obesity risk.

In the cross-sectional study, PC2-Z was associated with a higher risk for obesity and with higher BMI percentile and TG levels. Interestingly, the amino acid signature was associated significantly or as a trend (*P* < 0.1) with hepatic enzyme levels (ALT, AST and GGT). Increased BCAA serum levels have been observed in adults with non-alcoholic fatty liver disease (NAFLD)^27^, possibly by contributing to hepatic mitochondrial dysfunction^28^. Given that accurate, noninvasive biomarkers of liver disease in children are still needed^29^, and although we did not assess NAFLD in our children, it would be of interest to test whether this PC2 amino acid signature could add to improve prediction scores for NAFLD and other liver diseases.

Serum BCAA and AA levels have also been associated with future insulin resistance and dyslipidemia^13, 16, 30, 31^. Our longitudinal cohort demonstrated that the amino acid signature was not only associated with future BMI percentile but also with future hypertriglyceridemia risk, independently of gender and baseline BMI percentile, age and TG levels. These results are consistent with a recent 7-year follow up study of 396 girls where serum leucine levels predicted hypertriglyceridemia in early adulthood^32^. Interestingly in our longitudinal study 14% of the children developed hypertriglyceridemia in just 2 years, in contrast to the 13% developed in Finnish girls in seven years, highlighting the need of early biomarkers to prevent this disease. Our results show that not only leucine but a cluster of amino acids can predict hypertriglyceridemia in a much shorter time span, in both boys and girls.

Although, functionally it has not been established whether higher serum amino acids are biologically involved in the development of hypertriglyceridemia in humans. A recent study in rodents suggests that increased serum amino acid levels may have a causal role^33^. This study demonstrated that hepatic amino acid signaling regulates systemic lipid metabolism via a neuronal pathway, decreasing adipose lipoprotein lipase expression, thus suppressing plasma TG-hydrolysis activity and raising circulating TG levels. In the present study, the lack of association of the amino acid signature with serum ApoB, the major apolipoprotein contained in VLDL particles, is consistent with decreased TG clearance by peripheral tissues, rather than with higher TG exportation from the liver. Although further studies are need to fully elucidate the mechanism behind this effect, these amino acids could represent new targets for the prevention of dyslipidemia in children and ultimately future cardiovascular disease.

Circulating BCAA concentrations have been previously associated with future insulin resistance in children^12, 32^. However, while we observed an association of amino acids (PC2-Z) with HOMA-IR in the case control and cross-sectional studies, in the longitudinal study the association with future HOMA-IR did not reach statistical significance. This result could be confounded by effects of pubertal growth, as Tanner staging in children aged 9–11 has been associated with a decrease in insulin sensitivity^34^. Indeed, a limitation of our study is that Tanner staging was not assessed, so this result should be interpreted with caution.

Our study has other limitations that should be acknowledged. The diet of most of the participants was not assessed, and thus it was not possible to evaluate whether certain nutrients contribute to the serum amino acid levels variation. Moreover, it has been suggested that gut microbiota could also affect serum amino acid levels^35^, and thus should be included in the design of future studies. Finally, our study used a targeted metabolomics approach, and non-targeted approaches are still required to identify new metabolites associated with obesity and obesity-related complications.

The primary strength of this study is that, to the best of our knowledge, it is the first to include a large sample of children using tree different study designs, including validation in a longitudinal cohort. Moreover, this approach identified a metabolomics signature associated with obesity, which also showed predictive value for hypertriglyceridemia in Mexican children. It is important to mention that before attempting to translate these results into precision or personalized medicine; certain points need to be addressed. In addition to replication studies, pre-analytical methods need to be standardized, and normal levels of individual metabolites should be established in different biofluids, including total blood, plasma and serum.

In conclusion, this study shows that the amino acid signature composed of arginine, leucine/isoleucine, phenylalanine, tyrosine, valine and proline is associated with obesity, and is an independent risk factor and predictor of future hypertriglyceridemia in children.

## Electronic supplementary material

Supplemental information
