# Supplementary material for: An Amino Acid Signature Associated with Obesity Predicts 2-Year Risk of Hypertriglyceridemia in School-Age Children
Source: Sci Rep. 2017 Jul 17;7:5607. doi: 10.1038/s41598-017-05765-4 (PMC5514079; doi:10.1038/s41598-017-05765-4)
Supplement: Supplementary file 1 — Supplemental information [file 41598_2017_5765_MOESM1_ESM.pdf]

ONLINE SUPPLEMENTAL MATERIAL

An amino acid signature associated with obesity predicts 2-year risk of hypertriglyceridemia in school-age children

Sofia Moran-Ramos[1,2], Elvira Ocampo-Medina[2], Ruth Gutierrez-Aguilar[3,4], Luis Macías-Kauffer[2], Hugo Villamil-Ramírez[2], Blanca E. López-Contreras[2], Paola León-Mimila[2], Joel Vega-Badillo[2], Roxana Gutierrez-Vidal[2], Ricardo Villarruel-Vazquez[2], Erandi Serrano-Carbajal[2], Blanca E Del-Río-Navarro[3], Adriana Huertas-Vázquez[5], Teresa Villarreal-Molina[6], Isabel Ibarra-Gonzalez[7], Carlos A. Aguilar-Salinas[9], Marcela Vela-Amieva[8], Samuel Canizales-Quinteros[2]*

[1]Consejo Nacional de Ciencia y Tecnología (CONACYT), Mexico City, México.

[2]Unidad de Genómica de Poblaciones Aplicada a la Salud, Facultad de Química, UNAM/Instituto Nacional de Medicina Genómica (INMEGEN), Mexico City, México.

[3]Hospital Infantil México Federico Gómez, Mexico City, México.

[4]Facultad de Medicina, UNAM, Mexico City, México.

[5]Department of Medicine, David Geffen School of Medicine, UCLA, USA.

[6]Laboratorio de Enfermedades Cardiovasculares, INMEGEN, Mexico City, México.

[7]Instituto de Investigaciones Biomédicas, UNAM - Instituto Nacional de Pediatría, Mexico City, México.

[8]Laboratorio de Errores Innatos del Metabolismo y Tamiz, Instituto Nacional de Pediatría, Mexico City, México.

[9]Departamento de Endocrinología y Metabolismo, Instituto Nacional de Ciencias Médicas y Nutrición Salvador Zubirán, Mexico City, México.

Supplemental Table 1. Abbreviations and internal standard used for measured analytes by FIA-MS/MS

Analyte	Name	Internal Standard
Ala	Alanine	$^{2}H_{4}$-Ala
Arg	Arginine	$^{2}H_{4}$,^{13}C-Arg
Cit	Citrulline	$^{2}H_{2}$-Cit
Gly	Glycine	^{2}N,2-^{13}C-Gly
Leu/Ileu	Leucine/Isoleucine	$^{2}H_{3}$-Leu
Met	Methionine	$^{2}H_{3}$-Met
Orn	Ornithine	$^{2}H_{6}$-Orn
Phe	Phenylalanine	$^{13}C_{6}$-Phe
Pro	Proline	$^{13}C_{5}$-Pro
Tyr	Tyrosine	$^{13}C_{5}$-Tyr
Val	Valine	$^{2}H_{8}$-Val
C0	Free carnitine	$^{2}H_{9}$-C0
C2	Acetyl carnitine	$^{2}H_{3}$-C2
C3	Propionyl carnitine	$^{2}H_{3}$-C3
C3DC/C4OH	Malonyl/3-hydroxy-butyryl carnitine	$^{2}H_{3}$-C4
C4	Butyryl carnitine	$^{2}H_{3}$-C4
C4DC/C5OH	Methylmalonyl/3-hydroxy-isovaleryl carnitine	$^{2}H_{3}$-C5
C5	Isovaleryl carnitine	$^{2}H_{9}$-C5
C5:1	Tiglyl carnitine	$^{2}H_{9}$-C5
C5DC/C6OH	Glutaryl/3-Hydroxy-hexanoyl carnitine	$^{2}H_{6}$-C5DC
C6	Hexanoyl carnitine	$^{2}H_{3}$-C6
C6DC	Adipyl carnitine	$^{2}H_{6}$-C5DC
C8	Octanoyl carnitine	$^{2}H_{3}$-C8
C8:1	Octenoyl carnitine	$^{2}H_{3}$-C8
C10	Decanoyl carnitine	$^{2}H_{3}$-C10
C10:1	Decenoyl carnitine	$^{2}H_{3}$-C10
C10:2	Decadienoyl carnitine	$^{2}H_{3}$-C10
C12	Dodecanoyl carnitine	$^{2}H_{3}$-C12
C12:1	Dodecenoyl carnitine	$^{2}H_{3}$-C12
C14	Tetradecanoyl (Myristoyl) carnitine	$^{2}H_{3}$-C14
C14:1	Tetradecenoyl carnitine	$^{2}H_{3}$-C14
C14:2	Tetradecadienoyl carnitine	$^{2}H_{3}$-C14
C14OH	3-Hydroxy-tetradecanoyl carnitine	$^{2}H_{3}$-C14
C16	Hexadecanoyl (Palmitoyl) carnitine	$^{2}H_{3}$-C16
C16:1	Hexadecenoyl carnitine	$^{2}H_{3}$-C16
C16OH	3-Hydroxy-hexadecanoyl carnitine	$^{2}H_{3}$-C16

Supplemental Table 2. Characteristics of normal-weight and obese children from the case-control study.

	Normal-weight			Obese			*P* value
N (M/F)	311/295			305/209			**0.009**
		Percentile			Percentile		
	**Median**	**25**	**75**	**Median**	**25**	**75**	
**Clinical**							
Age (years)	9.9	8.3	11.3	10.1	8.6	11.5	**0.137**
Body weight (kg)	30	25	37	51	41	60	**$4.0x10^{-125}$**
Height (cm)	135	125	146	143	133	151	**$1.4x10^{-15}$**
BMI (kg/m ^{2})	16.4	15.6	17.4	24.6	22.9	26.7	**$6.0x10^{-183}$**
BMI percentile	47.6	30.0	59.6	97.4	96.2	98.5	**$2.6x10^{-183}$**
BMI z score	-0.06	-0.52	0.24	1.94	1.77	2.17	**$2.7x10^{-183}$**
Fat mass (% of BW)	25.3	21.9	29.35	45.25	41.78	48.8	**$1.5x10^{-170}$**
Lean mass (% of BW)	74.7	70.7	78.1	54.8	51.2	58.2	**$1.5x10^{-170}$**
Systolic blood pressure percentile	32.5	14.5	55.2	58.0	34.6	81.5	**$2.0x10^{-32}$**
Diastolic blood pressure percentile	63.7	42.7	81.3	67.7	46.0	85.7	**0.006**
**Biochemical**							
Glucose (mg/dL)	89	85	95	91	86.75	96	**0.001**
Insulin (μ U/mL)	4.4	3.0	6.3	10.6	6.8	15.5	**$7.5x10^{-81}$**
CRP (mg/dL)	0.027	0.01	0.076	0.202	0.092	0.389	**$6.5x10^{-69}$**
HOMA-IR	0.97	0.66	1.45	2.34	1.52	3.47	**$1.4x10^{-78}$**
Creatinine (mg/dL)	0.46	0.39	0.54	0.49	0.43	0.57	**$1.1x10^{-5}$**
Uric acid (mg/dL)	4.5	4	5.2	5.5	4.7	6.3	**$7.4x10^{-42}$**
**Lipids**							
Triacylglycerol (mg/dL)	74	54	103	129	94	175	**$4.9x10^{-66}$**
Total cholesterol (mg/dL)	166	147	182	177	155	197	**$1.4x10^{-9}$**
HDL-cholesterol (mg/dL)	51	45	59	41	35	47	**$2.9x10^{-54}$**
LDL-cholesteron (mg/dL)	97	82	111	106	91	121	**$1.2x10^{-9}$**
Apo-B	71	61	82	82	71	95	**$2.0x10^{-20}$**
**Liver enzymes**							
AST (U/L)	29	25	33	30	25	36	**0.007**
ALT (U/L)	18	15	21	26	20	38	**$5.1x10^{-61}$**
GGT (UL)	13	11	16	19	15	22	**$2.7x10^{-61}$**

Significant P-values are shown in bold

Supplemental Table 3. Association of principal components with biochemical parameters in normal-weight and obese children.

Clinical

BMI percentile

	All individuals				Normal-weight				Obese			
	β	t	P value	Adjusted *P*	β	t	P value	Adjusted *P*	β	t	P value	Adjusted *P*
PC1	0.87	3.84	**0.0001**	**0.0010**	-0.12	-0.66	0.51	0.68	0.02	1.07	0.29	0.48
PC2	5.08	14.64	**1.6x10^{-44}**	**5.12x10^{-42}**	0.86	2.51	**0.01**	**0.048**	0.04	1.46	0.15	0.30
PC3	0.87	1.83	0.07	0.17	0.49	1.15	0.25	0.44	0.04	1.44	0.15	0.31
PC4	0.11	0.20	0.83	0.90	0.28	0.62	0.54	0.69	0.00	-0.06	0.96	0.98
PC5	-3.37	-5.79	**9.4x10^{-9}**	**2.2x10^{-7}**	-0.51	-1.20	0.23	0.42	0.05	0.99	0.32	0.51
PC6	-1.54	-2.08	**0.04**	0.11	-0.50	-0.79	0.43	0.62	-0.04	-0.90	0.37	0.56

Body fat %

	All individuals				Normal-weight				Obese			
	β	t	P value	Adjusted *P*	β	t	P value	Adjusted *P*	β	t	P value	Adjusted *P*
PC1	0.48	5.35	**1.1x10^{-7}**	**2.2x10^{-6}**	0.14	2.17	**0.03**	0.10	0.11	1.72	0.09	0.21
PC2	1.87	13.32	**1.3x10^{-37}**	**2.7x10^{-35}**	0.25	1.98	**0.05**	0.14	0.01	0.10	0.92	0.97
PC3	0.29	1.52	**0.13**	**0.28**	-0.06	-0.37	0.71	0.83	0.05	0.39	0.70	0.82
PC4	0.17	0.79	0.42	0.61	0.51	3.12	**0.002**	**0.01**	-0.14	-0.97	0.33	0.53
PC5	-1.48	-6.39	**2.5x10^{-10}**	**7.6x10^{-9}**	-0.33	-2.21	**0.03**	0.09	-0.18	-0.88	0.38	0.57
PC6	-0.39	-1.30	0.19	0.37	0.42	1.81	0.07	0.18	-0.18	-0.89	0.37	0.57

Systolic blood

	All individuals				Normal-weight				Obese			
	β	t	P value	Adjusted *P*	β	t	P value	Adjusted *P*	β	t	P value	Adjusted *P*
PC1	-0.02	-1.20	0.23	0.42	-0.08	-2.97	**0.003**	**0.02**	0.04	1.46	0.15	0.30
PC2	0.08	2.57	**0.01**	**0.04**	0.05	1.02	0.31	0.50	0.10	2.21	**0.03**	0.09
PC3	0.20	5.22	**2.2x10^{-7}**	**4.1x10^{-6}**	0.28	4.46	**9.9x10^{-6}**	**0.0001**	0.13	2.69	**0.01**	**0.03**
PC4	-0.12	-2.89	**0.004**	**0.02**	-0.22	-3.64	**0.0003**	**0.002**	-0.02	-0.41	0.68	0.81
PC5	-0.01	-0.24	0.81	0.89	0.03	0.36	0.72	0.83	-0.07	-0.85	0.39	0.59
PC6	-0.10	-1.81	0.07	0.18	-0.23	-2.72	**0.007**	**0.03**	0.05	0.65	0.51	0.68

Diastolic blood

	All individuals				Normal-weight				Obese			
	β	t	P value	Adjusted *P*	β	t	P value	Adjusted *P*	β	t	P value	Adjusted *P*
PC1	-1.76	-0.08	0.94	0.97	-63.92	-2.13	**0.03**	0.10	77.71	2.28	**0.02**	0.08
PC2	78.70	1.98	**0.047**	0.14	87.07	1.61	0.11	0.24	38.25	0.64	0.52	0.68
PC3	115.50	2.44	**0.01**	0.06	152.03	2.06	**0.04**	0.12	72.85	1.17	0.24	0.43
PC4	36.66	0.72	0.47	0.64	26.29	0.37	0.71	0.83	61.75	0.86	0.39	0.59
PC5	-38.21	-0.57	0.57	0.72	-88.68	-1.04	0.30	0.49	90.13	0.78	0.44	0.62
PC6	66.49	0.97	0.33	0.53	8.40	0.09	0.93	0.97	169.89	1.75	0.08	0.20

Biochemical

Glucose

	All individuals				Normal-weight				Obese			
	β	t	P value	Adjusted *P*	β	t	P value	Adjusted *P*	β	t	P value	Adjusted *P*
PC1	-0.0003	-0.38	0.70	0.83	-0.0011	-1.00	0.32	0.51	0.0005	0.51	0.61	0.76
PC2	0.0063	4.91	**1.0x10^{-6}**	**1.9x10^{-5}**	0.0070	3.69	**0.0002**	**0.002**	0.0064	3.71	**0.0002**	**0.002**
PC3	-0.0006	-0.41	0.68	0.81	-0.0014	-0.53	0.60	0.74	0.0001	0.07	0.94	0.97
PC4	0.0020	1.24	0.22	0.41	0.0017	0.68	0.50	0.67	0.0023	1.07	0.29	0.48
PC5	-0.0042	-1.92	0.06	0.15	-0.0058	-1.93	**0.05**	0.15	-0.0040	-1.17	0.24	0.43
PC6	-0.0012	-0.54	0.59	0.74	0.0010	0.28	0.78	0.87	-0.0034	-1.19	0.24	0.42

Insulin

	All individuals				Normal-weight				Obese			
	β	t	P value	Adjusted *P*	β	t	P value	Adjusted *P*	β	t	P value	Adjusted *P*
PC1	-0.013	-2.55	**0.01**	**0.04**	-0.022	-3.00	**0.002**	**0.01**	-0.011	-1.60	0.11	0.25
PC2	0.088	9.77	**1.4x10^{-21}**	**7.9x10^{-20}**	0.097	7.31	**1.2x10^{-12}**	**4.7x10^{-11}**	0.076	6.47	**2.4x10^{-10}**	**7.61x10^{-9}**
PC3	0.003	0.24	0.81	0.89	-0.001	-0.04	0.97	0.98	-0.006	-0.46	0.65	0.79
PC4	0.037	3.20	**0.001**	**0.008**	0.072	4.11	**4.6x10^{-5}**	**0.0004**	0.014	0.95	0.34	0.53
PC5	-0.013	-0.76	0.45	0.62	-0.006	-0.24	0.81	0.89	-0.030	-1.25	0.21	0.40
PC6	-0.028	-1.76	0.08	0.19	-0.036	-1.51	0.13	0.28	-0.015	-0.77	0.44	0.62

CRP

	All individuals				Normal-weight				Obese			
	β	t	P value	Adjusted *P*	β	t	P value	Adjusted *P*	β	t	P value	Adjusted *P*
PC1	0.013	1.09	0.27	0.47	-0.012	-0.62	0.54	0.69	0.017	1.20	0.23	0.42
PC2	0.032	1.54	0.12	0.27	0.031	0.88	0.38	0.57	0.010	0.40	0.69	0.82
PC3	0.008	0.35	0.73	0.84	-0.014	-0.32	0.75	0.86	0.009	0.35	0.73	0.84
PC4	-0.070	-2.67	**0.01**	**0.034**	-0.079	-1.69	0.09	0.22	-0.048	-1.63	0.10	0.24
PC5	0.072	1.74	0.08	0.20	0.087	1.36	0.17	0.34	0.059	1.21	0.23	0.42
PC6	-0.063	-1.80	0.07	0.18	-0.119	-1.98	**0.05**	0.14	-0.032	-0.81	0.42	0.61

HOMA-IR

	All individuals				Normal-weight				Obese			
	β	t	P value	Adjusted *P*	β	t	P value	Adjusted *P*	β	t	P value	Adjusted *P*
PC1	-0.014	-2.53	**0.01**	**0.045**	-0.023	-2.99	**0.002**	**0.015**	-0.012	-1.61	0.11	0.24
PC2	0.094	10.02	**1.5x10^{-22}**	**1.5x10^{-20}**	0.103	7.39	**5.7x10^{-13}**	**3.0x10^{-11}**	0.081	6.74	**4.6x10^{-11}**	**1.7x10^{-9}**
PC3	0.001	0.11	0.91	0.97	-0.004	-0.23	0.82	0.89	-0.006	-0.46	0.65	0.79
PC4	0.040	3.31	**0.0009**	**0.006**	0.076	4.09	**5.1x10^{-5}**	**0.0005**	0.017	1.09	0.28	0.47
PC5	-0.018	-0.95	0.34	0.53	-0.011	-0.42	0.68	0.81	-0.033	-1.34	0.18	0.35
PC6	-0.027	-1.65	0.10	0.23	-0.032	-1.27	0.20	0.39	-0.017	-0.83	0.41	0.60

Creatinine

	All individuals				Normal-weight				Obese			
	β	t	P value	Adjusted *P*	β	t	P value	Adjusted *P*	β	t	P value	Adjusted *P*
PC1	-0.0019	-3.34	**8.6x10^{-4}**	**0.005**	-0.0018	-2.36	**0.02**	0.07	-0.0017	-1.98	**0.049**	0.14
PC2	0.0003	0.26	0.79	0.88	-0.0011	-0.77	0.44	0.62	0.0011	0.77	0.44	0.62
PC3	0.0056	4.69	**3.1x10^{-6}**	**4.6x10^{-5}**	0.0055	2.89	**0.004**	**0.02**	0.0052	3.43	**0.0006**	**0.005**
PC4	-0.0037	-2.92	**0.003**	**0.018**	-0.0057	-3.14	**0.002**	**0.01**	-0.0018	-1.04	0.30	0.49
PC5	-0.0013	-0.76	0.45	0.62	-0.0037	-1.69	0.09	0.22	0.0033	1.16	0.25	0.43
PC6	-0.0005	-0.27	0.79	0.88	-0.0024	-0.96	0.34	0.53	0.0027	1.11	0.27	0.46

Uric acid

	All individuals				Normal-weight				Obese			
	β	t	P value	Adjusted *P*	β	t	P value	Adjusted *P*	β	t	P value	Adjusted *P*
PC1	0.0030	4.79	**1.9x10^{-6}**	**3.1x10^{-5}**	0.0041	4.82	**1.9x10^{-6}**	**3.1x10^{-5}**	0.0013	1.43	0.15	0.31
PC2	-0.0035	-3.16	**0.002**	**0.009**	-0.0056	-3.69	**0.0002**	**0.002**	-0.0021	-1.33	0.19	0.36
PC3	0.0048	3.58	**0.0004**	**0.003**	0.0065	3.12	**0.002**	**0.01**	0.0027	1.63	0.10	0.24
PC4	-0.0066	-4.68	**3.2x10^{-6}**	**4.6x10^{-5}**	-0.0084	-4.20	**3.1x10^{-5}**	**0.0003**	-0.0053	-2.76	**0.006**	**0.028**
PC5	0.0023	1.21	0.23	0.42	-0.0001	-0.04	0.97	0.98	0.0052	1.67	0.10	0.22
PC6	0.0066	3.43	**0.0006**	**0.004**	0.0086	3.14	**0.002**	**0.01**	0.0061	2.36	**0.019**	0.07

Lipids													
Triacylglycerol		All individuals				Normal-weight				Obese			
		β	t	P value	Adjusted *P*	β	t	P value	Adjusted *P*	β	t	P value	Adjusted *P*
	PC1	-0.008	-2.05	**0.04**	0.12	-0.025	-4.69	**3.5×10^{-6}**	**4.8×10^{-5}**	0.007	1.21	0.23	0.42
	PC2	0.067	10.15	**3.4×10^{-23}**	**4.5×10^{-21}**	0.071	7.75	**4.1×10^{-14}**	**2.0×10^{-12}**	0.062	6.33	**5.5×10^{-10}**	**1.6×10^{-8}**
	PC3	0.001	0.15	0.88	0.95	-0.013	-0.96	0.34	0.53	0.006	0.60	0.55	0.70
	PC4	0.030	3.38	**0.0008**	**0.005**	0.072	5.90	**6.1×10^{-9}**	**1.5×10^{-7}**	-0.006	-0.47	0.64	0.78
	PC5	-0.022	-1.91	0.06	0.15	-0.009	-0.61	0.54	0.70	-0.045	-2.28	**0.02**	0.08
	PC6	-0.038	-3.17	**0.001**	**0.009**	-0.045	-2.61	**0.009**	**0.04**	-0.031	-1.86	0.06	0.17

otal cholesterol		All individuals				Normal-weight				Obese			
		β	t	P value	Adjusted *P*	β	t	P value	Adjusted *P*	β	t	P value	Adjusted *P*
	PC1	0.0013	0.85	0.40	0.59	0.0002	0.12	0.91	0.96	0.0024	1.02	0.31	0.50
	PC2	0.0073	2.76	**0.006**	**0.028**	0.0066	1.80	0.07	0.18	0.0079	1.97	**0.049**	0.14
	PC3	0.0069	2.15	**0.03**	0.10	0.0141	2.84	**0.005**	**0.02**	0.0025	0.59	0.55	0.71
	PC4	0.0002	0.05	0.96	0.98	0.0010	0.21	0.83	0.90	-0.0002	-0.04	0.97	0.98
	PC5	-0.0038	-0.84	0.40	0.59	-0.0021	-0.37	0.71	0.83	-0.0054	-0.69	0.49	0.66
	PC6	-0.0020	-0.43	0.66	0.80	-0.0039	-0.59	0.56	0.71	-0.0007	-0.10	0.92	0.97

DL-cholesterol		All individuals				Normal-weight				Obese			
		β	t	P value	Adjusted *P*	β	t	P value	Adjusted *P*	β	t	P value	Adjusted *P*
	PC1	-0.0004	-0.33	0.74	0.85	0.0011	0.66	0.51	0.68	-0.0015	-0.80	0.42	0.61
	PC2	-0.0042	-1.96	**0.05**	0.14	-0.0019	-0.63	0.53	0.69	-0.0039	-1.25	0.21	0.40
	PC3	0.0038	1.48	0.14	0.29	0.0086	2.14	**0.03**	0.10	0.0026	0.78	0.44	0.62
	PC4	0.0040	1.45	0.15	0.30	0.0010	0.26	0.80	0.88	0.0060	1.58	0.11	0.25
	PC5	-0.0024	-0.67	0.50	0.67	-0.0086	-1.85	0.06	0.17	0.0022	0.37	0.71	0.83
	PC6	0.0064	1.74	0.08	0.20	0.0003	0.05	0.96	0.98	0.0106	2.09	**0.04**	0.11

DL-cholesterol		All individuals				Normal-weight				Obese			
		β	t	P value	Adjusted *P*	β	t	P value	Adjusted *P*	β	t	P value	Adjusted *P*
	PC1	0.0152	1.40	0.16	0.32	0.0145	1.04	0.30	0.49	0.0137	0.78	0.44	0.62
	PC2	0.0026	0.13	0.89	0.95	0.0029	0.11	0.91	0.97	-0.0008	-0.03	0.98	0.98
	PC3	0.0395	1.79	0.07	0.18	0.0936	2.74	**0.006**	**0.029**	0.0084	0.29	0.77	0.87
	PC4	-0.0456	-1.91	0.06	0.15	-0.0612	-1.84	0.07	0.17	-0.0237	-0.68	0.50	0.67
	PC5	0.0316	1.01	0.31	0.51	0.0394	0.99	0.32	0.51	0.0249	0.45	0.65	0.79
	PC6	-0.0152	-0.47	0.64	0.78	-0.0125	-0.28	0.78	0.87	-0.0203	-0.44	0.66	0.80

Apo B		All individuals				Normal-weight				Obese			
		β	t	P value	Adjusted *P*	β	t	P value	Adjusted *P*	β	t	P value	Adjusted *P*
	PC1	-0.0037	-1.67	0.10	0.22	-0.0098	-2.74	**0.006**	**0.029**	-0.0008	-0.27	0.79	0.88
	PC2	0.0042	1.06	0.29	0.49	0.0085	1.33	0.18	0.36	0.0004	0.07	0.94	0.97
	PC3	0.0041	0.92	0.36	0.55	0.0126	1.58	0.11	0.25	-0.0002	-0.04	0.97	0.98
	PC4	0.0001	0.02	0.99	0.99	0.0036	0.41	0.68	0.81	-0.0002	-0.04	0.97	0.98
	PC5	0.0088	1.14	0.25	0.44	0.0111	0.95	0.34	0.53	0.0071	0.69	0.49	0.66
	PC6	0.0006	0.08	0.93	0.97	-0.0085	-0.76	0.44	0.62	0.0063	0.74	0.46	0.63

Liver enzymes													
AST		All individuals				Normal-weight				Obese			
		β	t	P value	Adjusted *P*	β	t	P value	Adjusted *P*	β	t	P value	Adjusted *P*
	PC1	-0.0007	-3.66	**0.0002**	**0.002**	-0.0004	-1.79	0.07	0.18	-0.0008	-2.61	**0.009**	**0.04**
	PC2	0.0007	2.12	**0.03**	0.10	0.0013	3.43	**0.0006**	**0.004**	0.0002	0.39	0.70	0.82
	PC3	-0.0013	-3.17	**0.001**	**0.009**	0.0003	0.47	0.64	0.78	-0.0021	-3.71	**0.0002**	**0.002**
	PC4	-0.0009	-2.20	**0.03**	0.09	-0.0003	-0.57	0.57	0.72	-0.0015	-2.28	**0.02**	0.08
	PC5	0.0002	0.30	0.77	0.87	0.0004	0.59	0.56	0.71	0.0001	0.07	0.94	0.97
	PC6	-0.0014	-2.39	**0.02**	0.06	-0.0014	-1.99	**0.047**	0.14	-0.0012	-1.32	0.19	0.37

ALT		All individuals				Normal-weight				Obese			
		β	t	P value	Adjusted *P*	β	t	P value	Adjusted *P*	β	t	P value	Adjusted *P*
	PC1	-0.0005	-3.30	**0.0009**	**0.006**	-0.0002	-1.16	0.25	0.44	-0.0005	-2.54	**0.01**	**0.05**
	PC2	0.0002	0.94	0.35	0.54	0.0011	3.38	**0.0007**	**0.005**	-0.0004	-1.08	0.28	0.48
	PC3	-0.0005	-1.51	0.13	0.28	0.0000	0.08	0.93	0.97	-0.0006	-1.53	0.13	0.28
	PC4	0.0001	0.29	0.77	0.87	0.0001	0.24	0.81	0.89	0.0001	0.14	0.89	0.95
	PC5	0.0001	0.28	0.78	0.87	0.0003	0.51	0.61	0.76	-0.0001	-0.16	0.87	0.93
	PC6	0.0002	0.53	0.60	0.74	-0.0005	-0.76	0.45	0.62	0.0009	1.50	0.13	0.28

GGT		All individuals				Normal-weight				Obese			
		β	t	P value	Adjusted *P*	β	t	P value	Adjusted *P*	β	t	P value	Adjusted *P*
	PC1	-0.0009	-2.67	**0.008**	**0.03**	0.0011	2.49	**0.01**	**0.05**	-0.0016	-3.18	**0.002**	**0.009**
	PC2	-0.0003	-0.50	0.61	0.76	-0.0005	-0.70	0.48	0.66	-0.0009	-0.97	0.33	0.53
	PC3	-0.0032	-4.58	**5.1×10^{-6}**	**6.6×10^{-5}**	-0.0021	-2.14	**0.03**	0.10	-0.0039	-4.17	**3.6×10^{-5}**	**3.9×10^{-4}**
	PC4	0.0001	0.19	0.85	0.92	0.0009	0.95	0.34	0.53	0.0003	0.28	0.78	0.87
	PC5	0.0001	0.09	0.93	0.97	0.0023	2.04	**0.04**	0.12	-0.0005	-0.29	0.77	0.87
	PC6	-0.0006	-0.63	0.53	0.69	0.0008	0.56	0.58	0.72	-0.0007	-0.46	0.65	0.79

The beta value is given for the Box-Cox transformed variables. Insulin, CRP, ApoB, glucose, TG, total cholesterol and HOMA-IR were log-transformed. Creatinine and fat mass were square root-transformed. Other variables were transformed according to the following λ factors: λ=2 for diastolic blood pressure percentile, λ=1 for ALT, λ=-0.5 for GGT and AST, and λ=0.25 for uric acid and HDL cholesterol. The multivariate model was adjusted by gender, age and BMI percentile. P-values were corrected by Fdr and significant values are shown in bold. Significant P-values are shown in bold.

Supplemental Table 4. Characteristics of the cross-sectional study of Mexican children.

	All subjects (554) M/F (278/276)		
		Percentile	
	**Median**	**25**	**75**
**Clinical**			
Age (years)	10.0	8.4	11.5
Body weight (kg)	36.0	27.3	45.0
Height (cm)	138.2	127.4	147.3
BMI (kg/m2)	19.0	16.9	21.1
BMI percentile	81.3	60.1	90.9
BMI z score	0.89	0.22	1.34
Fat mass (% of BW)	33.8	25.1	40.4
Lean mass (% of BW)	66.2	59.5	74.9
Systolic blood pressure percentile	38.0	21.4	58.3
Diastolic blood pressure percentile	75.5	59.7	86.0
**Biochemical**			
Glucose (mg/dL)	91	86	96
Insulin (µ U/mL)	5.7	4	8.8
CRP (mg/dL)	0.03	0.01	0.10
HOMA-IR	1.28	0.87	1.97
Creatinine (mg/dL)	0.47	0.41	0.54
Uric acid (mg/dL)	4.7	4.1	5.6
**Lipids**			
Triacylglycerol (mg/dL)	86	61	121
Total cholesterol (mg/dL)	166	148	187
HDL-cholesterol (mg/dL)	49	41	57
LDL-cholesteron (mg/dL)	98	83	113
**Liver enzymes**			
AST (U/L)	30	26	34
ALT (U/L)	19	16	24
GGT (UL)	14	12	17

Supplemental Table 5. Effect of PC2-Z on metabolic traits in the cross-sectional study of Mexican children.

	All subjects (554) M/F (278/276)			
	**β**	**t**	***P* value**	**Adjusted *P***
**Clinical**				
BMI percentile	0.899	3.089	**0.002**	**0.011**
Fat mass (% of BW)	0.388	4.089	**$5.0x10^{-5}$**	**0.0006**
Systolic blood pressure percentile	-0.004	-0.219	0.827	0.879
Diastolic blood pressure percentile	-1.096	-0.049	0.961	0.980
**Biochemical**				
Glucose (mg/dL)	-0.002	-2.022	**0.044**	0.104
Insulin (µ U/mL)	0.014	2.698	**0.007**	**0.036**
CRP (mg/dL)	-0.025	-2.485	**0.014**	0.057
HOMA-IR	0.013	2.334	**0.020**	0.066
Creatinine (mg/dL)	0.000	-0.634	0.526	0.711
Uric acid (mg/dL)	0.001	1.936	0.053	0.116
**Lipids**				
Triacylglycerol (mg/dL)	0.017	4.324	**$1.8x10^{-5}$**	**0.0003**
Total cholesterol (mg/dL)	0.002	1.429	0.153	0.240
HDL-cholesterol (mg/dL)	-0.005	-2.589	**0.010**	**0.045**
LDL-cholesteron (mg/dL)	0.013	1.159	0.247	0.363
**Liver enzymes**				
AST (U/L)	0.000	-2.313	**0.021**	0.066
ALT (U/L)	0.000	-2.233	**0.026**	0.076
GGT (U/L)	-0.001	-4.937	**$1.1x10^{-6}$**	**$4.9x10^{-5}$**

The beta value is given for the Box-Cox transformed variables. Insulin, ApoB, glucose, TG, total cholesterol, HDL-cholesterol and HOMA-IR were log-transformed. Creatinine, systolic blood pressure percentile and LDL-cholesterol were square root-transformed. Other variables were transformed according to the following λ factors: λ=2 for diastolic blood pressure percentile, λ=-1 for ALT, λ=-0.9 for GGT, λ=-0.5 for AST, λ=-0.25 for CRP and λ=0.25 for uric acid. The association of hepatic enzymes and CRP with PC2-Z is positive, however due to the nature of the transformation (λ=-x) the β appears negative. The multivariate model was adjusted by gender, age and BMI percentile. Significant P-values are shown in bold.

Supplemental Table 6. Effect of PC2-Z on metabolic traits in the cross-sectional study of Mexican children stratified by weight status.

	Underweight (41)			Normal-weight (285)			Overweight (177)			Obese (51)		
**Clinical**	**β**	***P* value**	**Adjusted P**	**β**	***P* value**	**Adjusted P**	**β**	***P* value**	**Adjusted P**	**β**	***P* value**	**Adjusted P**
Fat mass (% of BW)	0.026	0.146	0.431	0.022	0.056	0.270	0.018	0.142	0.431	0.025	0.196	**0.494**
Systolic blood pressure percentile	0.066	0.246	0.519	-0.014	0.641	0.822	-0.019	0.589	0.813	-0.073	0.227	0.519
Diastolic blood pressure percentile	8.083	0.919	0.961	7.921	0.811	0.915	-35.957	0.424	0.740	8.408	0.917	0.961
**Biochemical**												
Glucose (mg/dL)	-0.001	0.706	0.842	-0.001	0.242	0.519	-0.002	0.165	0.449	-0.001	0.600	0.813
Insulin (μU/mL)	-0.014	0.614	0.813	0.009	0.263	0.521	0.019	**0.041**	0.252	0.031	**0.053**	0.270
CRP (mg/dL)	-0.038	0.571	0.813	0.001	0.945	0.972	-0.054	**0.002**	0.064	-0.021	0.622	0.813
HOMA-IR	-0.016	0.604	0.813	0.006	0.424	0.740	0.018	**0.048**	0.270	0.030	0.063	0.285
Creatinine (mg/dL)	-0.001	0.596	0.813	0.000	0.804	0.915	-0.001	0.252	0.519	-0.001	0.673	0.832
Uric acid (mg/dL)	0.001	0.751	0.881	0.001	0.492	0.780	0.002	0.137	0.431	0.002	0.493	0.780
**Lipids**												
Triacylglycerol (mg/dL)	0.000	0.966	0.972	0.015	**$9.9x10^{-3}$**	0.096	0.023	**$6.7x10^{-3}$**	0.096	0.033	**0.025**	0.168
Total cholesterol (mg/dL)	0.004	0.493	0.780	0.000	0.865	0.949	0.005	0.144	0.431	-0.001	0.821	0.915
HDL-cholesterol (mg/dL)	0.002	0.706	0.842	-0.004	0.152	0.432	-0.007	0.080	0.334	-0.015	**0.024**	0.168
LDL-cholesterol (mg/dL)	0.026	0.444	0.755	0.001	0.972	0.972	0.032	0.176	0.461	-0.044	0.412	0.740
**Liver enzymes**												
AST (U/L)	0.000	0.414	0.740	0.000	0.661	0.832	0.000	0.268	0.521	-0.002	**0.008**	0.096
ALT (U/L)	0.000	0.911	0.961	0.000	0.214	0.519	0.000	0.518	0.782	-0.001	**0.005**	0.096
GGT (U/L)	-0.001	0.507	0.782	-0.001	**0.009**	0.096	-0.001	**0.022**	0.168	-0.002	**0.000**	**0.023**

The beta value is given for the Box-Cox transformed variables. Insulin, ApoB, glucose, TG, total cholesterol, HDL-cholesterol and HOMA-IR were log-transformed. Creatinine, systolic blood pressure percentile and LDL-cholesterol were square root-transformed. Other variables were transformed according to the following λ factors: λ=2 for diastolic blood pressure percentile, λ=-1 for ALT, λ=-0.9 for GGT, λ=-0.5 for AST, λ=-0.25 for CRP and λ=0.25 for uric acid. The association of hepatic enzymes and CRP with PC2-Z is positive, however due to the nature of the transformation (λ=-x) the β appears negative. The multivariate model was adjusted by gender, age and BMI percentile. Significant P-values are shown in bold.

Supplemental Table 7. Characteristics of Mexican children in the longitudinal cohort, at baseline and after 2 years.

	All subjects T0 (301) M/F (137/164)			All subjects T1			
		Percentile			Percentile		
	**Median**	**25**	**75**	**Median**	**25**	**75**	***P* value**
**Clinical**							
Age (years)	8.7	7.3	9.8	10.7	9.3	11.9	
Body weight (kg)	31	24	39	40	32	49	**$8.8x10^{-50}$**
Height (cm)	131	123	138	143	135	151	**$1.4x10^{-50}$**
BMI (kg/m2)	17.6	15.9	20.7	19.5	17.0	22.8	**$9.1x10^{-35}$**
BMI percentile	77.8	47.2	95.0	80.6	47.9	94.5	0.632
BMI z score	0.76	-0.09	1.61	0.87	-0.05	1.59	0.789
Fat mass (% of BW)	31.0	24.4	38.2	35.6	26.6	41.6	**$4.6x10^{-18}$**
Lean mass (% of BW)	69.0	61.8	75.7	64.4	58.4	73.4	**$1.7x10^{-18}$**
Systolic blood pressure percentile	56	31	81	34	15	62	**$1.3x10^{-11}$**
Diastolic blood pressure percentile	64	45	82	63	43	85	0.846
**Biochemical**							
Glucose (mg/dL)	90	85	95	89	84	95	**0.046**
Insulin (μ U/mL)	5.0	3.0	7.8	6.4	4.2	10.0	**$1.9x10^{-10}$**
CRP (mg/dL)	0.06	0.03	0.17	0.06	0.02	0.19	0.562
HOMA-IR	1.12	0.64	1.73	1.42	0.86	2.32	**$4.0x10^{-9}$**
Creatinine (mg/dL)	0.80	0.40	1.00	0.48	0.43	0.54	**$8.4x10^{-13}$**
Uric acid (mg/dL)	5.0	4.0	5.5	5.1	4.3	5.9	**$1.5x10^{-11}$**
**Lipids**							
Triacylglycerol (mg/dL)	92	59	131	94	61	133	0.543
Total cholesterol (mg/dL)	175	155	193	167	149	183	**$8.2x10^{-7}$**
HDL-cholesterol (mg/dL)	48	40	57	47	40	56	**0.02**
LDL-cholesterol (mg/dL)	104	87	121	97	83	113	**$2.5x10^{-8}$**
Apo-B	77	67	87	77	65	88	0.837
**Liver enzymes**							
AST (U/L)	29	26	34	29	24.5	33	**0.049**
ALT (U/L)	19	15	22	20	17	26.5	**$9.5x10^{-7}$**
GGT (UL)	13	11	16	15	12	19	**$2.5x10^{-10}$**

Significant P-values are shown in bold

Supplemental Table 8. Effect of baseline PC2-Z on future metabolic traits in Mexican children of the longitudinal cohort.

	**All subjects T0 (301) M/F (137/164)**			**Adjusted *P***
**Clinical**	**β**	**t**	***P* value**	
BMI percentile	2.377	4.640	**5.3x10^{-6}**	**5 x10^{-5}**
Fat mass (% of BW)	0.950	5.293	**2.4x10^{-7}**	**4.5x10^{-6}**
Systolic blood pressure percentile	0.058	1.377	0.170	0.248
Diastolic blood pressure percentile	0.804	1.888	0.060	0.127
**Biochemical**				
Glucose (mg/dL)	0.003	1.709	0.089	0.153
Insulin (μ U/mL)	0.026	2.245	**0.026**	0.081
CRP (mg/dL)	0.029	1.183	0.238	0.282
HOMA-IR	0.018	1.837	0.067	0.128
Creatinine (mg/dL)	-0.002	-1.242	0.215	0.273
Uric acid (mg/dL)	0.009	2.467	**0.014**	0.054
**Lipids**				
Triacylglycerol (mg/dL)	0.038	4.327	**2.1x10^{-5}**	**1.3x10^{-4}**
Total cholesterol (mg/dL)	0.007	2.144	0.033	0.089
HDL-cholesterol (mg/dL)	-0.006	-1.402	0.162	0.248
LDL-cholesteron (mg/dL)	0.150	0.316	0.752	0.752
Apo-B	0.000	-1.264	0.207	0.273
**Liver enzymes**				
AST (U/L)	0.008	1.987	**0.048**	0.114
ALT (U/L)	-0.001	-1.100	0.272	0.287
GGT (U/L)	-0.001	-1.143	0.254	0.284

The beta value is given for the Box-Cox transformed variables. CRP, insulin, glucose, TG, uric acid, TG, total cholesterol, HDL-cholesterol and AST were log-transformed. HOMA-IR and systolic blood pressure percentile were square root-transformed. Other variables were transformed according to the following λ factors: λ=-0.5 for ApoB, ALT and GGT. The remainder variables were normally distributed. The multivariate model was adjusted by gender, age and BMI percentile. Significant P-values are shown in bold.

Supplemental Table 9. Associations between baseline amino acids levels and hypertriglyceridemia

		95 % Confidence interval		
**Amino acid**	**OR**	**Lower CI**	**Upper CI**	**P value**
Arginine	1.73	0.97	3.14	0.0650
Leucine/Isoleucine	1.86	1.05	3.3	**0.0300**
Phenylalanine	2.12	1.21	3.81	**0.0094**
Tyrosine	1.37	0.82	2.31	0.2281
Valine	1.61	0.94	2.77	0.0778
Proline	0.976	0.59	1.62	0.9272

Regression adjusted for age, sex, sampling calendar year, baseline BMIpercentile, HOMA and serum TG
